# Primary graft failure associated with epithelial downgrowth: a case report

**DOI:** 10.1186/1471-2415-5-11

**Published:** 2005-05-25

**Authors:** Anthony J Aldave, David A Hollander, Bruno Branco, Brooks Crawford, Richard L Abbott

**Affiliations:** 1 From the Department of Ophthalmology, The University of California, San Francisco/ Francis I. Proctor Foundation, San Francisco, California, USA; 2 The Jules Stein Eye Institute, The University of California, Los Angeles, Los Angeles, California, USA

## Abstract

**Background:**

Epithelial downgrowth is a rare complication of ocular surgery. While the features of epithelial downgrowth following corneal transplantation are well described, its association with primary graft failure has only been reported once previously. We report a case of primary corneal graft failure (PGF) associated with retrocorneal epithelial cell ingrowth.

**Case presentation:**

A 59 year-old male underwent an uncomplicated penetrating keratoplasty for Fuchs' corneal dystrophy. The patient developed PGF, and a second transplant was performed 5 weeks after the initial surgery. The initial host corneal button and the failed corneal graft were examined with light microscopy. Histopathologic examination of the excised corneal button demonstrated multilaminar epithelial cells on the posterior corneal surface and absence of endothelial cells. DNA extraction and polymerase chain reaction (PCR) for herpes simplex virus (HSV) DNA was performed on the failed corneal graft. Polymerase chain reaction performed on the failed corneal graft was negative for HSV DNA, which has been implicated in selected cases of PGF. Three years following repeat penetrating keratoplasty, there was no evidence of recurrent epithelial ingrowth.

**Conclusion:**

This is only the second report of PGF associated with epithelialization of the posterior corneal button, which most likely developed subsequent to, instead of causing, the diffuse endothelial cell loss and primary graft failure.

## Background

Epithelial downgrowth is an unusual but recognized complication of ocular surgery [1]. Characteristic features of epithelial downgrowth following corneal transplantation are well described [2], although the association of epithelial downgrowth and PGF has only been made once previously [3]. We present the second case of PGF in which the excised corneal button demonstrated epithelialization of the posterior cornea, most likely secondary to postoperative epithelial cell migration across a denuded Descemet's membrane.

## Case presentation

A 59 year-old male with Fuchs' endothelial dystrophy presented with visually limiting pseudophakic corneal edema in his right eye with a best-corrected visual acuity of 20/50. Cataract extraction had been performed two years previously in the right eye through a 3.2 mm limbal incision. An uncomplicated penetrating keratoplasty was performed, with an 8.25 mm donor cornea secured into an 8 mm recipient opening with 16 interrupted 10-0 nylon sutures. On the first postoperative day, the graft demonstrated diffuse 3^+^–4^+ ^stromal edema and a nearly complete epithelial defect. The anterior chamber was deep, and no aqueous leakage was noted from either the wound or suture tracks. The epithelial defect resolved in the first postoperative week, although 1^+^–2^+ ^stromal edema persisted. As the graft remained edematous 4 weeks later, the patient was diagnosed with PGF, and a repeat penetrating keratoplasty was performed 5 weeks after the primary graft. Three years later, the second graft remains clear, with a central pachymetry of 495 μm, and corrected visual acuity of 20/25+.

**Figure 1:**
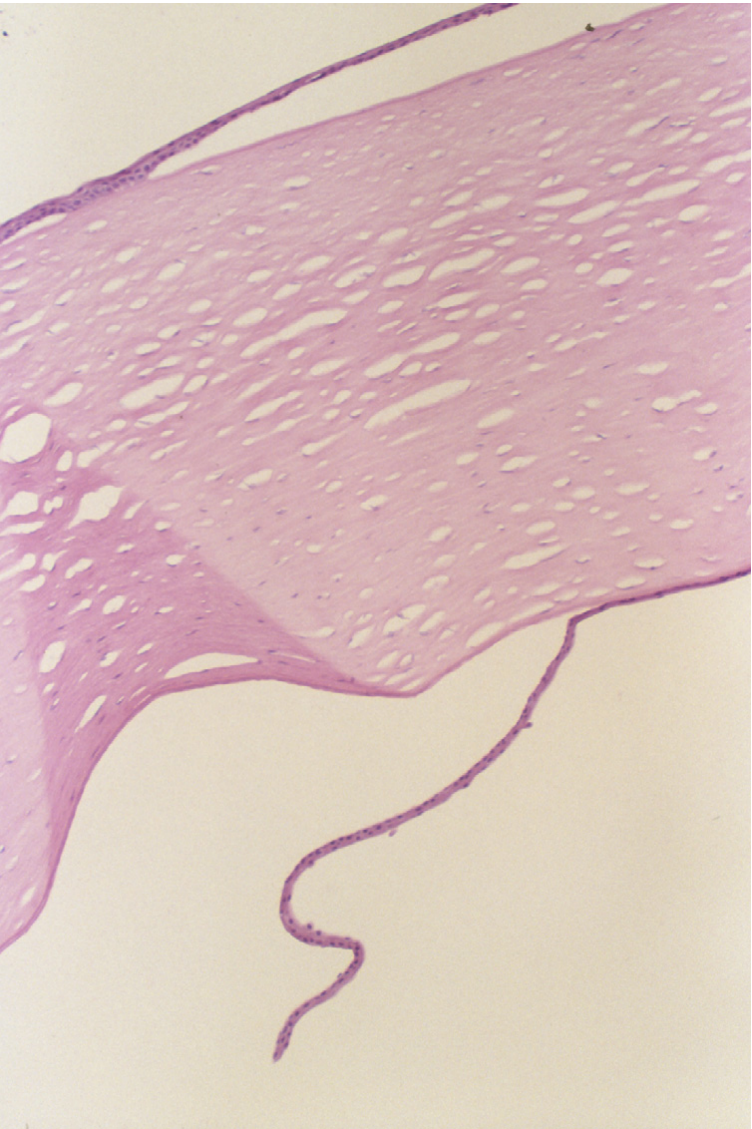
Histopathologic section of excised corneal button demonstrating a markedly thickened, edematous stroma and the presence of multilayered epithelial cells on the anterior and posterior surfaces (hematoxylin and eosin stain, original magnification × 50).

**Figure 2:**
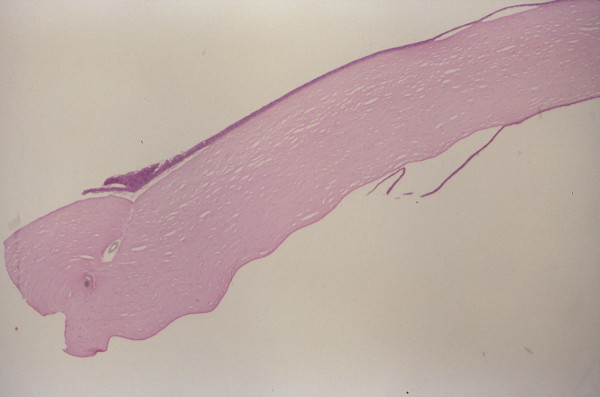
Histopathologic section of excised corneal button demonstrating anterior portion of suture track seen as focal tissue separation on superior side of corneal button. The identification of Descemet's membrane as a dark band on the inferior side of the corneal button indicates that the donor corneal button had been correctly oriented when secured into the recipient opening (periodic acid Schiff stain, original magnification × 50).

The host corneal button removed at the initial penetrating keratoplasty and the failed donor corneal button were formalin-fixed and analyzed with light microscopy after staining with hematoxylin and eosin, periodic acid-Schiff, and Masson trichrome stains. Histopathologic examination of the host corneal button revealed stromal edema, thickening and excrescences of Descemet's membrane, and a decreased number of endothelial cells, consistent with Fuchs' corneal dystrophy. Histopathologic examination of the failed corneal graft revealed a markedly thickened stroma and near complete absence of endothelial cells. Both sides of the excised donor corneal button were covered with epithelial cells, consistent with epithelial downgrowth. (Figure 1) The corneal sections containing suture tracks were stained with the periodic acid Schiff stain to demonstrate Descemet's membrane. (Figure 2) The suture tracks passed through the true epithelial layer, Bowman's layer, and extended into the stroma, confirming that the button had not been inverted when secured into the recipient opening. Additionally, serial sectioning of the host corneal button failed to demonstrate the presence of epithelial cells on the posterior corneal surface.

To investigate the possibility of HSV infection of the donor cornea, which has been associated with one-third of cases of PGF [4,5], PCR analysis of the donor corneal button for HSV DNA was performed. Positive and negative controls confirmed adequate extraction of purified DNA from the failed donor corneal button, as well as the sensitivity and specificity of the HSV primers used. PCR analysis for HSV DNA was negative in the donor corneal button.

A complete review of the first donor's medical and eye bank records was performed. The tissue was obtained through the University of California, San Francisco tissue bank from a 56 year-old female donor who died from a myocardial infarction and cardiac tamponade. Routinely performed serologic screening tests and biomicroscopic examination of the donor button were unremarkable. The endothelial cell count was 2852 cells/mm^2^, and death to preservation was 12 hours, with a 51-hour preservation time in Optisol-GS.

The paired donor cornea was transplanted into another recipient several hours after this patient's original transplant. This cornea also appeared unremarkable on biomicroscopic examination, remained in Optisol-GS for 53 hours prior to transplantation, and had a cell count of 2841 cells/mm^2^. Five months later, after a similar 16 interrupted suture technique, the graft was clear and compact with central corneal pachymetry of 538 μm.

## Conclusion

Epithelial downgrowth is an unusual complication after penetrating keratoplasty [1-3,6-17]. (Table 1) The incidence in patients undergoing aphakic penetrating keratoplasty has been estimated at 0.25% [3], and risk factors include poor wound apposition, full-thickness suture passes, and globe hypotony [1]. Characteristic signs, such as a migratory endothelial line, elevated intraocular pressure and effacement of the normal iris architecture, have been reported between 14 days and 13 years after penetrating keratoplasty [2,3].

The histopathologic finding of epithelial downgrowth on the posterior corneal surface after a diagnosis of PGF is rare. Sugar and colleagues reported a patient with PGF 1 week after penetrating keratoplasty [3]. Histopathologic examination of the failed corneal graft excised 2 weeks after the initial transplant demonstrated stratified squamous epithelium covering Descemet's membrane. Though the patient had undergone a previous uncomplicated intracapsular cataract extraction, there was no evidence of epithelial downgrowth on the patient's native cornea. The authors speculated that epithelial migration over Descemet's membrane had occurred during storage.

Identifying the origin of the epithelial cells (donor or host), as well as the timing of epithelial cell access to the posterior cornea, in our case required further investigation. Inadvertent inversion of the donor corneal button during penetrating keratoplasty was excluded by identifying suture tracks extending into the stroma from the true anterior corneal surface.

If PGF had occurred secondary to epithelial cell migration over Descemet's membrane, the migration would have had to begin while the donor cornea was in storage medium. Since epithelial migration is inhibited by an intact endothelium, epithelial extension would have required diffuse endothelial cell loss [18]. In this case, there was no evidence of endothelial loss during harvesting. Additionally, there was no evidence of HSV infection of the donor cornea.

Storage at 4°C, as in the case we report, is designed to inhibit microbial cellular metabolism, and we are unaware of any evidence that human corneal epithelial cells retain sufficient metabolic activity at 4°C to allow proliferation and migration. Furthermore, the donor button in this case was preserved for only 51 hours prior to transplantation, an insufficient amount of time for the epithelial cells to have migrated over the scleral rim and onto the posterior corneal surface [19].

If the epithelial cells on the posterior corneal surface were not of donor origin, then they must be host-derived. The possibility that these cells gained entry during the prior cataract surgery was dismissed by both the absence of epithelial cells on the original host button and the lack of evidence of epithelial downgrowth prior to penetrating keratoplasty.

The most likely explanation is that epithelial cells from the peripheral host cornea rim gained access to the posterior corneal surface after the graft was secured into the recipient opening. The complete absence of endothelial cells on the excised donor button indicates that the PGF occurred secondary to a diffuse endothelial cell injury while the corneoscleral button was in the preservative medium or at the time of penetrating keratoplasty. Interestingly, none of the identified risk factors for epithelial downgrowth, such as wound dehiscence, hypotony or a shallow anterior chamber, were observed.

## Methods

### DNA extraction

A 20 milligram formalin-fixed, paraffin-embedded tissue section from the failed corneal donor button was deparaffinized using xylene, and then treated with alcohol to remove the residual xylene. Protein digestion was performed with 40 μl of 20mg/ml proteinase K at 55°C for 12 hours. A silica-gel-membrane technology (Qiagen, Valencia, CA) was used to isolate 50 μl of purified DNA product.

### Polymerase chain reaction

Polymerase chain reaction (PCR) for herpes simplex virus (HSV) I DNA was performed on the failed donor button, with a number of simultaneously performed positive and negative controls. Positive controls consisted of varied amounts of HSV I viral DNA (1, 10, 100 and 1000 copies), allowing quantification of the assay's sensitivity to HSV DNA present in the donor corneal button. DNA extracted from the cornea of a patient with keratoconus served as a negative control.

A previously described primer set was used for amplification of a 92bp HSV sequence: forward, 5'- CATCACCGACCCGGAGAGGGAC and reverse, 5'- GGGCCAGGCGCTTGTTGGTGTA^1^. These are regions of identity in the genomes of HSV types 1 and 2, and do not discriminate between these virus types [20-22]. PCR amplification was performed on a 100 μl final volume containing: 1 μl of 19.7mM of each primer (Biomolecular Research Center, University of California, San Francisco), 5 μl of the extracted DNA product, 10 μl of a 10X PCR Buffer (Sigma Chemical, Saint Louis, MO), 2 μl of 10mM deoxy nucleotide triphosphate (dNTP) (PE Biosystems, Foster City, CA), 10 μl of 25 mM of magnesium chloride, 2.5 μl of RedTaq DNA polymerase (Sigma Chemical), and 68.5 μl of water. Thermal cycling was performed in a GeneAmp^® ^PCR System 9700 (Applied Biosystems, Foster City, CA) with the following program: initial denaturation for 2 min at 94°C, followed by 41 cycles of 94°C for 30 seconds, 60°C for 30 seconds, and 72°C for 30 seconds, followed by a final extension for 2 minutes at 72°C. The PCR amplification products (10 μl) were separated on a 4% polyacrylamide gel and visualized by ethidium bromide staining.

To confirm the presence of purified DNA extracted from the failed donor corneal button, previously published primers were used to perform PCR amplification of exon 12 of the *TGFBI *gene from the purified DNA product [23]. A mixture containing water in place of purified DNA served as a negative control.

## Competing interests

The author(s) declare that they have no competing interests.

## Authors' contributions

Anthony J. Aldave, M.D. Assisted with care of the patient, coordinated performance of PCR for HSV DNA, wrote majority of the manuscript.

David A. Hollander, M.D., M.B.A. Assisted with manuscript preparation and revisions.

Bruno Branco, M.D. Performed PCR for HSV DNA and wrote description of methods for Material and Methods section as well as relevant portion of Results section.

Brooks Crawford, M.D. Performed histopathologic analysis and wrote description of methods for Material and Methods section as well as relevant portion of Results section.

Richard L. Abbott, M.D. Primary ophthalmologist for the patient. Performed initial and subsequent corneal transplants.

## Pre-publication history

The pre-publication history for this paper can be accessed here:



## Figures and Tables

**Table 1:** Summary of Reported Cases of Epithelial Downgrowth (ED) after Penetrating Keratoplasty (PK)

Author	Previous ocular surgery	PK Indication	Time from PK to ED diagnosis	Clinical features of ED	Diagnosis of ED	Final Anatomic/ Visual Status
Chiou et al [1]	3 PKs, 2 Trabs, 1 CPC	Graft Failure	13 months	Concentric peripheral retrocorneal membrane	Confocal microscopy	BSCVA 20/400

Feder & Krachmer [2]	ICCE	ABK	6 months	Iritis, glaucoma, posterior corneal line	Paracentesis, laser	Graft decompensation, glaucoma
	ICCE	PBK	7 months	Iritis, glaucoma	Paracentesis, laser	End-stage glaucoma
	ICCE	Fuchs' dystrophy	5 months	Posterior corneal line	Paracentesis, laser	Evisceration
	ECCE	KCN	13 years	Iritis	Paracentesis, laser	Phthisis bulbi

Mazow [7]	None	Endothelial dystrophy	5 months	Glaucoma	Histopathologic	Enucleation

Karabatsas [8]	2 PKs	PBK	8 months	Posterior corneal line, glaucoma	Clinical features	Not available

Sugar [3]	PK, ICCE	Graft failure	7 months	Retrocorneal membrane, pre-iris membrane	Histopathologic	Recurrent retrocorneal membrane; CF VA*
	ICCE	ABK;Fuchs' dystrophy	2 weeks	Descemet's deposits	Histopathologic	BSCVA 20/200^†^
	LK, ICCE	Opacification of lamellar graft	16 months	Pre-iris membrane	Histopathologic	Scarred regraft; VA 10/400
	PK, ICCE	Graft failure	6 months	Retrocorneal membrane	Clinical features	Vascularized, edeamatous regraft, VA HM

Leibowitz [9]	2 PKs	Graft failure	1 month	Retrocorneal membrane; anterior chamber epithelial cyst	Histopathologic	Vascularized, opacified regraft

Groh & Naumann [10]	None	KCN	13 months	Anterior chamber epithelial cyst	Clinical features	VA 14/20
	None	Lattice corneal dystrophy	47 months	Anterior chamber epithelial cyst	Clinical features	VA 10/20

Wearne [11] ^‡^	None	Scarring 2° to HSV keratitis	10 years	Anterior chamber epithelial cyst	Clinical features	VA 6/20

Yamaguchi et al. [12]	PK, ICCE	Graft failure	12 months	Retrocorneal membrane	Clinical features and histopathologic	Opacified regraft

Sidrys [13] ^¶^	ICCE	PBK	3 weeks^§^	Retrocorneal membrane	Clinical features and histopathologic	VA 6/60^Θ^

Weiner [14]^#^	Unknown	Various	Unknown	Retrocorneal membrane, glaucoma	Clinical features and histopathologic	Unknown

Kurz & D'Amico [16]	None	Corneal scar	10 months	None	Histopathologic	Unknown
	None	Corneal scar	8 weeks	None	Histopathologic	Enucleation**

Bennett & D'Amico [15]	None	KCN	3 years	Iris epithelial inclusion cyst	Clinical features and histopathologic	S/p sector iridectomy without recurrence

Claoue et al [17]	None	KCN	21 months	Iris epithelial inclusion cyst	Clinical features and histopathologic	S/p sector iridectomy without recurrence

Daneshvar et al. [6]	CE	ABK	1 year	Glaucoma, retrocorneal membrane	Laser, ultrasound biomicroscopy	No recurrence 16 months after repeat PK

BSCVA = best spectacle corrected visual acuity; ABK = aphakic bullous keratopathy; CF = counting fingers; CPC = cyclophotocoagulation; ECCE = extracapsular cataract extraction; HM = hand motions; HSV = herpes simplex virus; ICCE = intracapsular cataract extraction; KCN = keratoconus; LK = lamellar keratectomy; PBK = pseudophakic bullous keratopathy; Trab = trabeculectomy; * limited by ARMD; ^† ^h/o CME and RD s/p SB; ^‡^same patient reported again in; 10^§ ^epithelial downgrowth through a cataract wound fistula; ^Θ ^limited by CME; ^¶ ^same patient reported again in; 12 ^# ^13 patients reported; ** performed after traumatic wound dehiscence and total retinal detachment
